# Primary leiomyosarcoma of the pancreas: report of a case treated by local excision and review of the literature

**DOI:** 10.1186/s40792-015-0097-2

**Published:** 2015-10-06

**Authors:** Anna Caterina Milanetto, Valbona Liço, Stella Blandamura, Claudio Pasquali

**Affiliations:** Pancreatic and Digestive Endocrine Surgical Unit—Department of Surgery, Gastroenterology and Oncology, University of Padua, Via Giustiniani, 2-35128 Padua, Italy; Pathology-Department of Medicine, University of Padua, Via Gabelli, 61-35128 Padua, Italy

**Keywords:** Pancreatic tumour, Leiomyosarcoma, Mesenchymal tumour, Sarcoma, Pancreas

## Abstract

**Background:**

First described by Ross in 1951, primary pancreatic leiomyosarcoma is a rare mesenchymal tumour of the pancreas, with nonspecific clinical and radiological features and a poor prognosis, if unresectable.

**Case report:**

A 60-year-old woman presented with abdominal pain. Magnetic resonance imaging (MRI) and computed tomography (CT) scan detected a dishomogeneous egg-shaped 8-cm mass, arising from the pancreatic head, with duodenal compression, without dilation of the Wirsung duct. ^18^F-FDG positron-emission tomography (PET)-CT showed a moderate tracer uptake, and the endoscopic ultrasound (US) showed a hypoechoic lesion, arising from the duodenal wall, suspected to be a gastrointestinal stromal tumour (GIST). CEA, CA19-9, NSE, and chromogranin A were normal. At the surgical exploration, a 10-cm mass, adherent to the anterior aspect of the pancreatic head, was found. The lesion was easily separable from the duodenal wall and was totally excised. The frozen intraoperative examination showed a mesenchymal tumour, with spindle-shaped cells, suggesting that a GIST diagnosis was likely. Postoperative course was uneventful. Histology and immunohistochemistry demonstrated a well-differentiated leiomyosarcoma, with five to six mitotic counts per 10 high-power field (HPF) and proliferative index (MIB-1) 10 % (grade 2 according to Federation Nationale des Centres de Lutte Contre le Cancer (FNCLCC)), with positive smooth muscle actin, desmin, and caldesmon but negative CD117 (c-kit) and S-100. The patient is alive and asymptomatic 19 months after surgery, without evidences of disease.

**Conclusions:**

In the English literature, only 44 cases of primary pancreatic leiomyosarcoma have been reported. If a pancreatic mass suspected for primary pancreatic leiomyosarcoma has no adjacent organ/vessel invasion or distant metastases, surgical resection is the therapy of choice.

## Background

The pancreas is mainly composed of an exocrine component, which includes acini and ducts, and an endocrine component, the islets of Langerhans, but stroma is scant in normal pancreas [1].

Ductal adenocarcinoma represents the most common primary tumour arising in the pancreas. Mesenchymal tumours may involve the pancreas, but most of them are secondary lesions from extra pancreatic tumours. Primary mesenchymal pancreatic tumours are very rare [2], with only about 200 cases reported in the English literature [3].

Leiomyosarcoma is the most frequent primary malignant mesenchymal tumour of the pancreas and represents the 0.1 % of malignant pancreatic tumours [4]. Primary pancreatic leiomyosarcoma was first described by Ross in 1951 [5], and until now, only 44 cases [3–34] have been reported in the English literature.

We reported a case of primary pancreatic leiomyosarcoma, which was treated with local excision.

## Case presentation

**Fig. 1:**
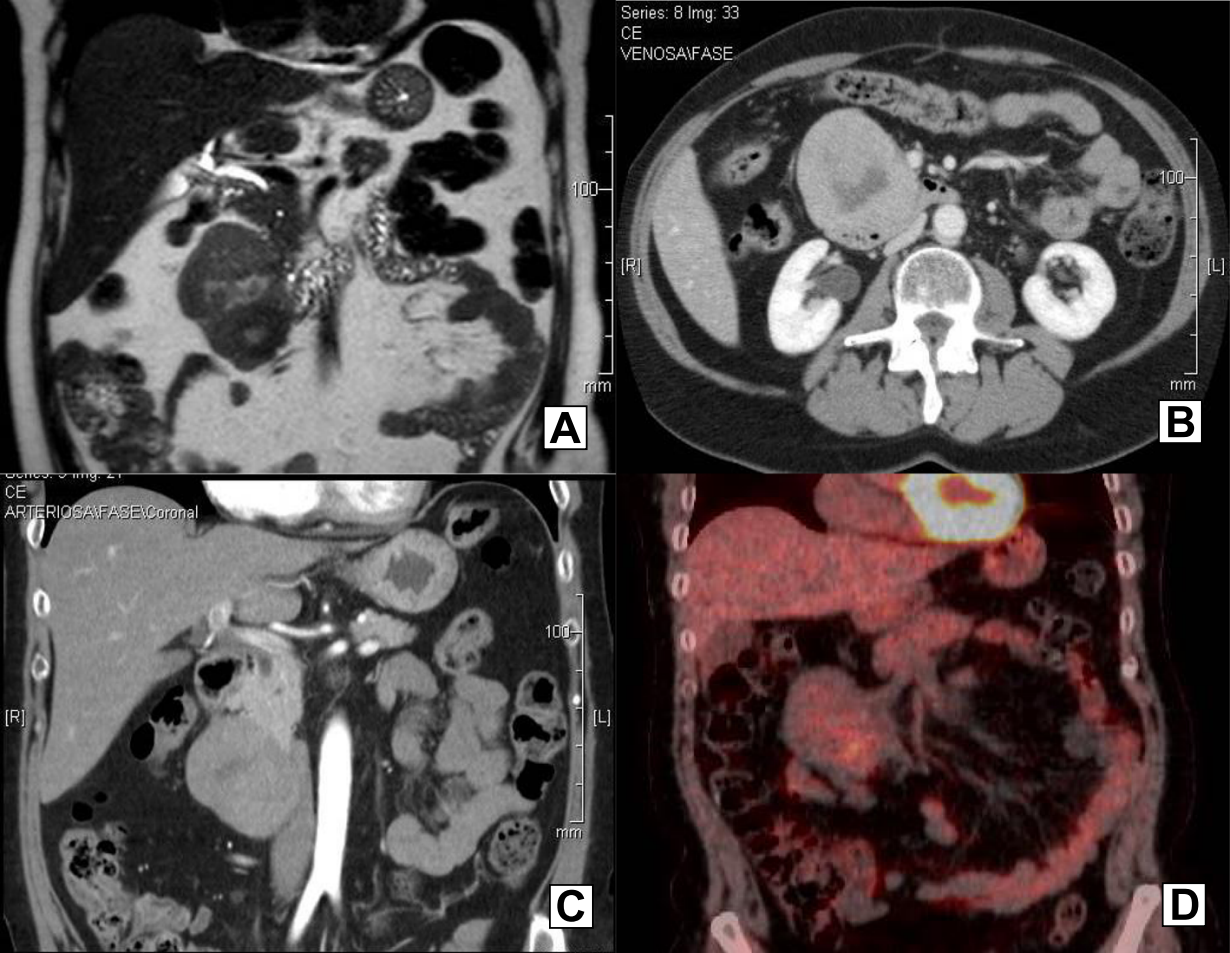
Pre-operative imaging. MRI scan: the dishomogeneous mass, arising from the pancreatic head (**a**). CT scan: no evidence of invasion of the surrounding tissues; CT venous phase: duodenal compression on the third duodenal part. (**b**) CT arterial phase: mass arising from the pancreatic head (**c**). ^18^F-FDG-PET-CT: moderate tracer uptake by the pancreatic lesion (**d**)

A 60-year-old woman complained abdominal pain since a few years, and she had several ultrasound (US) scans, which resulted negative. In November 2013, an abdominal magnetic resonance imaging (MRI) detected a dishomogeneous egg-shaped mass (main diameter 8 cm), arising from the lower part of the pancreatic head (Fig. 1a), with normal appearance of the main pancreatic duct.

The patient did not complain anorexia or weight loss but only dyspepsia, and physical examination was unremarkable. The pancreatic lesion was confirmed by a computed tomography (CT) scan. There was no evidence of invasion of the surrounding tissues (gastroduodenal wall, retroperitoneal connective tissue, and common bile duct) and no evidence of liver or lymph node involvement (Fig. 1b, 1c).

At the ^18^F-FDG positron-emission tomography (PET)-CT, the lesion showed only a moderate tracer uptake, suggesting a gastrointestinal stromal tumour (GIST) (Fig. 1d), and at the endoscopic US, the mass was hypoechoic and non-homogeneous, with well-defined margins, arising from the duodenal wall, proper to a GIST. Serum tumour markers CEA, CA19-9, NSE, and chromogranin A were normal.

In January 2014, the patient underwent surgery. The mass was adherent to the anterior surface of the pancreatic head and had a vascular peduncle, a branch-side of the anterior-inferior pancreatico-duodenal vein, that drained into the gastro-colic trunk of Henle.

**Fig. 2:**
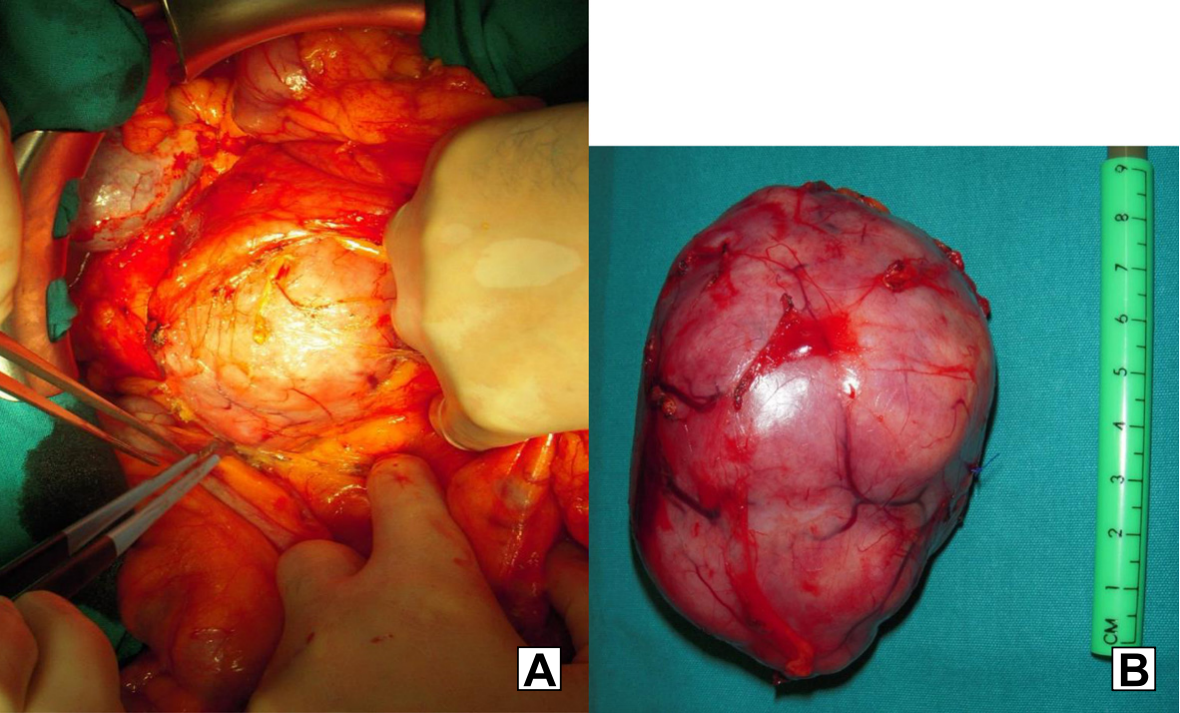
Intraoperative findings. The mass was adherent to the anterior surface of the pancreatic head, and it was easily dissociable from the duodenal wall (**a**). The 10-cm mass was totally excised. Macroscopically, it had a smooth and polylobulated surface (**b**)

The mass compressed the third duodenal part and was easily dissociable from the duodenal wall; then, it was totally excised, including the pancreatic capsule, without any pancreatic resections (Fig. 2a). A frozen analysis of the specimen showed a mesenchymal tumour, with spindle-shaped cells, suggesting that a GIST diagnosis was likely, and intraoperative US excluded other pancreatic lesions.

Postoperative course was uneventful, and the patient was discharged on postoperative day 4^th^.

Macroscopically, the lesion had a smooth and polylobulated surface, had a fasciculated internal appearance, and measured 10 cm as maximum diameter (Fig. 2b).

**Fig. 3:**
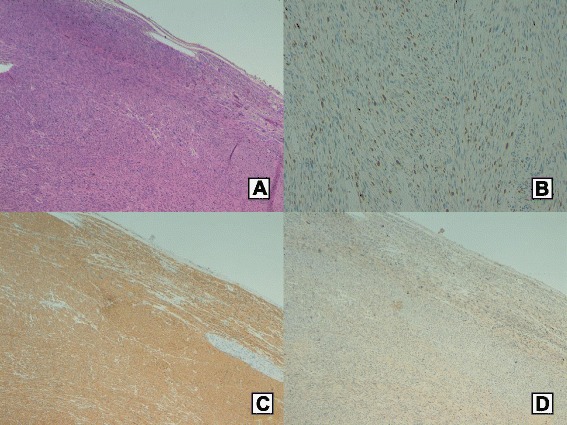
Histology and immunohistochemical analysis. Haematoxylin-eosin stain (original magnification ×50) (**a**). Mib1 (proliferative index) (**b**). Strong immunoreactivity to smooth muscle actin (**c**). No immunoreactivity to CD117 (c-kit) (**d**)

Histology revealed a well-differentiated leiomyosarcoma (Fig. 3a), composed by spindle cells with a fasciculated growth pattern, with five to six mitotic counts per 10 high-power field (HPF) and proliferative index (MIB-1) 10 % (grade 2 according to (FNCLCC) system [35]) (Fig. 3b). Surgical margins were negative, and no pancreatic tissue was included in the specimen.

Immunohistochemical analysis revealed positive smooth muscle actin (Fig. 3c), desmin, and caldesmon but negative CD117 (c-kit) (Fig. 3d) and S-100. Malignant epithelial elements were not identified, excluding a sarcomatoid carcinoma.

**Fig. 4:**
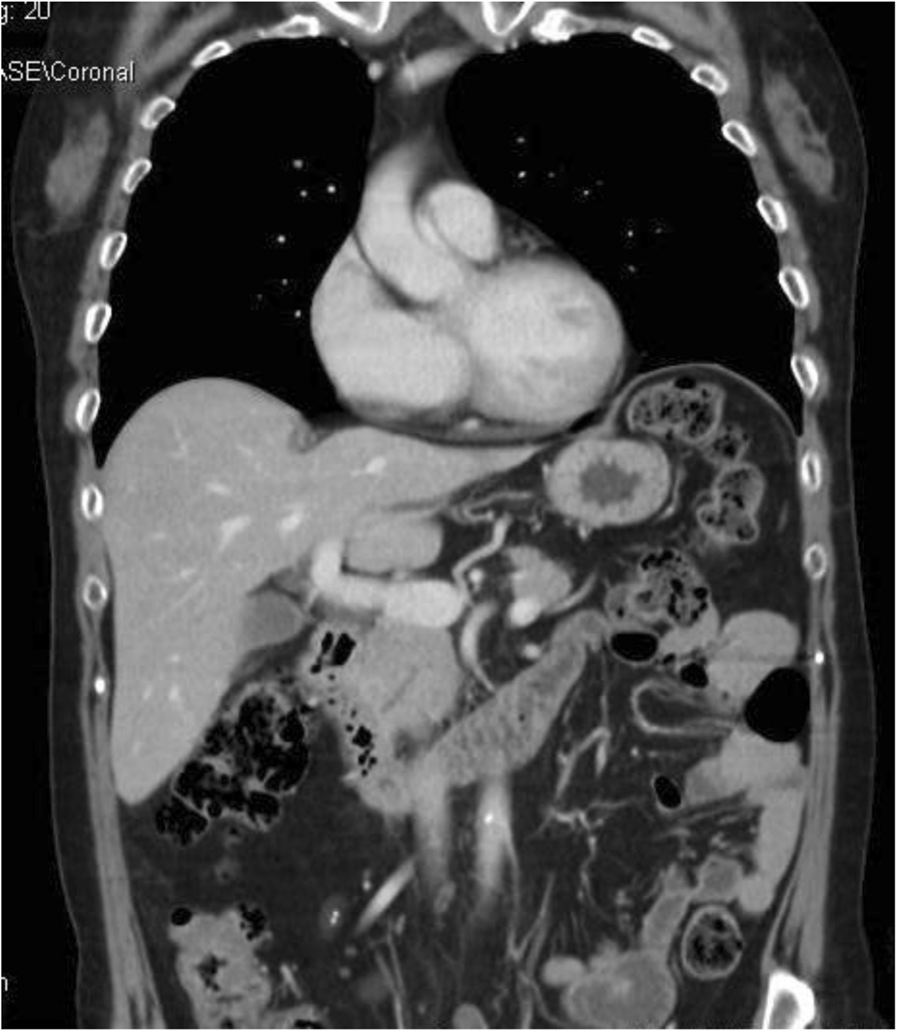
Postoperative imaging. Thorax-abdominal CT scan: normal appearance of the pancreatico-duodenal-biliary region, without any signs of liver or lung involvement

The patient did not receive postoperative adjuvant radio- or chemo-therapy. She is still alive and asymptomatic after 19 months of follow up (f.u.). The last thorax-abdominal CT performed did not detect any signs of disease recurrence (Fig. 4).

### Discussion

First described by Ross in 1951 [5], primary pancreatic leiomyosarcoma is considered to arise either from smooth muscle cells of the pancreatic ducts or from the wall of small intra-pancreatic vessels [7, 8, 22]. In our case, we can only speculate if the tumour originated from the pancreatic vessels, since we obtained negative surgical margins and we did not have pancreatic tissue around the excised tumour.

From our review of the English literature (Table 1) [3–34], a pancreatic leiomyosarcoma is more common in the fifth decade of life (range 14–87 years, median age 51 years) and in female patients (M = 20, F = 24). The tumour size is variable, ranging from 1 to 30 cm (median 8.0 cm), and it seems to be more frequently localised in the body-tail of the pancreas (*n* = 24). Abdominal pain, weight loss, and abdominal mass are the most commonly presenting symptoms, but these are nonspecific [36].

**Table 1:** Reported cases of primary pancreatic leiomyosarcoma in the English literature

Case	Author	Year	Age, year/sex	Site/size, cm	Distant metastasis	Treatment	Grade	IHC	Outcome/follow up, months
1	Ross [5]	1951	80/M	Whole pancreas	Widespread	Autopsy case	High	n.a.	DOD
2	Berman and Levene [6]	1956	47/M	Head/5.5	No	PD	n.a.	n.a.	ANED/12
3	Feinberg et al [7]	1957	14/M	Head/11.0	No	PD	Low	n.a.	n.a.
4–8	Baylor and Berg (*n* = 5) [4]	1973	51 (median)/3M, 2F	Body-tail/n.a.	1 localised, 1 locally advanced, 3 disseminated	n.a.	n.a.	n.a.	ANED (*n* = 1)/1; DOD (*n* = 3)/14 (median)
9	Ishikawa et al [8]	1981	44/M	Head/8.0	No	PD/CT	Low	n.a.	DOD/48
10	Lakhoo and Mannell [9]	1991	68/M	Body/17.0	No	DP, gastric resection, transverse colectomy	Low	n.a.	ANED/24
11	de Alava et al [10]	1993	71/M	Body/3.6	No	DP	n.a.	(+) desmin, smooth muscle actin, vimentin; (−) AE1/AE3, CAM5.2	n.a.
12	Peskova and Fried [11]	1994	68/F	Head/15.0	No	PD	Low	n.a.	ANED/36
13	Sato et al [12]	1994	53/F	Body/25.0	No	DP	n.a.	(+) desmin, smooth muscle actin; (−) S-100, EMA	n.a.
14	Ishii et al [13]	1994	66/M	Tail/4.5	Liver	Non resectable/CT	n.a.	(+) desmin, smooth muscle actin	DOD/33
15	Aranha et al [14]	1995	46/F	Body/3.0	No	DP/CT	High	(+) desmin; (−) S-100, cytokeratin, HMB-45	DOD/9
16	Owen et al [15]	1997	40/M	Head/7.0	No	PD	n.a.	(+) desmin, smooth muscle actin	ANED/120
17	Shimizu et al [16]	1997	49/F	Head/15.0	Lung	Non resectable/CT	n.a.	(+) desmin, smooth muscle actin; (−) S-100	DOD/3
18	Chawla et al [17]	1998	45/F	Head/9.2	Lung	Non resectable/CT	Low	(+) smooth muscle actin	AWD/19
19	Paciorek and Ross [18]	1998	63/F	Body/2.0	Mesentery, single	DP	n.a.	n.a.	ANED/n.a.
20	Zalatnai et al [19]	1998	57/M	Head/6.0	Liver	Non resectable	3	(+) smooth muscle actin, SMA; (−) S-100, desmin	DOD/7
21	Ferlan-Marolt et al [20]	2000	57/F	Body-tail/12.0	No	DP	2	(+) SMA; (−) desmin	DOC/p.o. period
22	Machado et al [21]	2000	52/M	Head/7.5	No	PD	Low	(+) SMA; (−) S-100	ANED/24
23	Deveaux et al [22]	2001	44/F	Head/5.0	No	PD	1	(+focal) smooth muscle actin; (−) S-100, keratin	ANED/48
24	Nesi et al [23]	2001	76/M	Tail/8.0	No	DP	High	(+) smooth muscle actin, SMA; (−) desmin, CD34, cytokeratin, S-100	DOD/12
25	Aihara et al [24]	2002	25/F	Body/3.5	No	Local excision	n.a.	(+) desmin, smooth muscle actin; (−) S-100	ANED/42
26	Komoda et al [25]	2002	52/F	Head/1.5	No	PD	Low	(+) desmin, smooth muscle actin; (−) S-100, CD34, KIT	ANED/12
27	Maarouf et al [26]	2007	40/F	Tail/5.0	No	DP	Low	(+) desmin, smooth muscle actin, H-caldesmon; (−) CD34	ANED/240
28	Muhammad et al [27]	2008	73/M	Body/10.0	Liver	Non resectable/CT	Low to intermediate	(+) desmin, smooth muscle actin; (−) S-100, CD34, cytokeratin AE1/AE3, HMB45	DOD/3
29-37	Zhang H et al [28] (*n* = 9)	2010	63 (median)/5M, 4F	Head (*n* = 7); Tail (*n* = 2)/8.0 (median)	Liver (*n* = 4)	PD (*n* = 4); Palliative/biopsy (*n* = 5)	Grades 4 (*n* = 1), 3 (*n* = 5), 2 (*n* = 3)	(+) smooth muscle actin, desmin; (−) KIT	DOD (*n* = 5), DOC/Unknown (*n* = 4)/13 (median)
38	Riddle et al [29]	2010	83/F	Tail/8.2	No	DP	2	(+) smooth muscle actin, desmin, vimentin; (−) c-kit, CD34, S-100, pan-cytokeratin	ANED/8
39	Zhang et al [30]	2011	56/F	Body-tail/13.0	No	DP	2	(+) desmin, smooth muscle actin, H-caldesmon, vimentin; (−) cytokeratin, CD34, S-100, CD117	ANED/14
40	Hur et al [31]	2011	70/F	Head/5.0	No	PD	2	(+) actin, vimentin, desmin; (−) cytokeratin, S-100, CD34, CD117	DOD/22
41	Izumi et al [32]	2011	41/F	Body/4.5	No	DP	n.a.	(+) smooth muscle actin, desmin, vimentin	ANED/14
42	Vanderpuye et al [33]	2011	59/F	Tail/24.0	Liver, transverse colon	DP/ CT + RT	n.a.	n.a.	AWD/24
43	Moletta et al [34]	2012	54/F	Body and tail/13.0	Liver	DP, left hepatectomy/CT	3	(+) smooth muscle actin, desmin; (−) cytokeratin, CD34, S-100, CD117	AWD/37
44	Kim et al [3]	2014	51/F	Tail/5.5	No	DP/RT	2	(+) desmin, SMA; (−) CD117, HMB45, CD34	ANED/27
45	*Present case*	2014	60/F	Head/10.0	No	Local excision	2	(+) smooth muscle actin, desmin, caldesmon;(−) S-100, CD117	ANED/19

*ANED* alive and no evidence of disease, *AWD* alive with disease, *cm* centimetres, *CT* chemotherapy, *DOC* died of other cause, *DOD* died of disease, *DP* distal pancreatectomy, *IHC* immunohistochemistry, *n* number of cases, *n.a.* not available, *PD* pancreatico-duodenectomy, *p.o.* postoperative, *RT* radiotherapy

No serum tumour marker is expected to be positive since the mesenchymal origin of the tumour.

Imaging studies are often unspecific [31]. In our review [13, 14, 18, 21–24, 27, 28, 30–32, 34], pancreatic leiomyosarcoma appears mainly as a heterogeneous enhancing mass. As the tumour volume increases, haemorrhagic, necrotic, and cystic changes, often associated to a highly aggressive behaviour, can be observed, and large pancreatic leiomyosarcoma with a cystic degeneration can be misdiagnosed as pseudocyst [12] or as cystadenocarcinoma [8].

In MRI, the unenhanced T1-weighted and T2-weighted images are most useful in tumour localization [18], and ^18^F-FDG-PET-CT is able to identify a lesion with aggressive behaviour, which may show an increased metabolic activity [21]. CT or US-guided fine-needle aspiration biopsy (FNAB) may help the diagnosis of those advanced or unresectable [13].

In our case, there was no evidence of surrounding tissue involvement or of distant disease localizations, and the pancreatic mass had a dishomogeneous appearance at MRI and CT scans, but the moderate tracer uptake at ^18^F-FDG-PET-CT and the endoscopic US features suggested a GIST diagnosis. We did not perform an EUS-guided biopsy because with our equipment, a FNA biopsy was not allowed. Differentiating the variety of mesenchymal tumours by imaging is extremely difficult, and only histology with immunohistochemical studies can define the final diagnosis.

At the histology, a primary leiomyosarcoma of the pancreas shows well-formed fascicles of spindle cells with blunt-ended nuclei intersecting at vertical angles, varying degrees of pleomorphism [23], abundant eosinophilic cytoplasm [28], and possible increased mitotic activity [3]. However, differential diagnosis includes other soft tissue tumours: inflammatory myofibroblastic tumour, non-myogenic spindle cell sarcoma (namely fibrosarcoma), solitary fibrous tumour, liposarcoma, rhabdomyosarcoma, undifferentiated pleomorphic sarcoma (so-called malignant fibrous histiocytoma), malignant peripheral nerve sheath tumour, and gastrointestinal stromal tumour [23, 28].

In immunohistochemical analysis, stromal tumours are usually diagnosed as myogenic (i.e. leiomyomas or leiomyosarcomas) when they are diffusely positive for desmin or smooth muscle actin, as neurogenic when positive for S-100 protein or as gastrointestinal stromal tumours (GIST) when positive for CD117 (c-kit) [37]. Leiomyosarcoma usually exhibits strong activity for muscle markers despite occasional keratin positivity. In our case, frozen section analysis was coherent with a GIST, but the final histological and immunohistochemical analyses demonstrated a leiomyosarcoma and excluded a sarcomatoid carcinoma and a GIST. Intraoperative frozen section is not able to define the variety of mesenchymal neoplasm; although it has a low reliability, it can exclude a carcinoma and therefore can be helpful in the surgical strategy.

Primary leiomyosarcoma of the pancreas has a high incidence of distant metastases and regional invasion, with rare lymphatic involvement [28], which could be the key point to differential diagnosis [36]. In our review of the English literature, out of the 44 cases reported, 16 had distant metastases at the time of diagnosis that mainly occurred into the liver (*n* = 9) and lung (*n* = 2).

Sharp demarcation of the growth appearing characteristic for this type of pancreatic neoplasm with no invasion into the surrounding organs enabled complete excision in spite of the large size of the tumour [20]. In this review, 28 patients benefit from a radical surgical treatment (pancreatico-duodenectomy and distal pancreatectomy, *n* = 27; local excision, *n* = 1). Twenty-three patients had only surgery and 13 of them are alive without any evidences of disease (median f.u. 24 months, range 2–240 months). Five patients also received adjuvant treatment (chemotherapy and/or radiotherapy) after surgery, and only one is alive without disease 27 months after surgery and radiotherapy treatment for a grade 2 tumour. Ten patients had unresectable disease: four of them received chemotherapy (three died of disease, mean overall survival 13 months), and six patients untreated or had only biopsy (median survival 13 months).

Radical resection of the lesion has been reported to be the best choice for cure of pancreatic leiomyosarcoma [14], and the benefit of adjuvant therapy after radical pancreatectomy is still unknown [8, 14, 28]. In our case, a local excision was performed; histology showed a grade 2 leiomyosarcoma, and the patient is alive without any evidences of disease 19 months after surgery, without having adjuvant therapy. In the English literature, there is only one case reporting a local excision for a 3.5-cm pancreatic leiomyosarcoma, alive without disease 42 months after surgery [24].

Although tumour size is an important indicator with regard to resectability, it does not seem to affect the clinical course after surgical resection [23, 36]. Xu et al. [36] showed with multivariate analysis that non-radical resection was an independent adverse prognostic factor. Mitotic counts of more than 10 mitoses per 10 HPF were reported as another adverse predictor [23]. In our review, grading (according to FNCLCC system [35]) was available in 29 out of 44 patients, and there were 17 low-grade (or grade 1, 2) lesions and 12 high-grade (or grade 3, 4) ones. Among the first group, 9 patients are alive without evidence of disease (median f.u. 27 months, range 8–240 months) and one is alive with disease after 19 months of f.u. Among the 12 high-grade patients, 9 died of disease (median overall survival 9.5 months, range 3–98 months).

## Conclusions

In the English literature only 44 cases of primary pancreatic leiomyosarcoma have been reported. The patients present mainly with abdominal pain, and at imaging, this tumour rarely presents a lymphatic spread. If a pancreatic mass suspected for primary leiomyosarcoma has no adjacent organ/vessels invasion or distant metastases, surgical resection is the therapy of choice. Resected patients have longer overall survival compared to unresectable ones. Operative specimen allows an accurate diagnosis that depends on histological and immunohistochemical analyses, and in unresectable cases, a biopsy is mandatory providing to perform immunohistochemistry. Unfortunately, before surgery or intraoperatively, no help comes from FNA-cytology or frozen section analysis in order to better distinguish among spindle cells tumours. The benefit of adjuvant therapies is still unproven.

## Consent

Written informed consent was obtained from the patient for publication of this case report and any accompanying images. A copy of the written consent is available for review by the Editor-in-Chief of this journal.
